# Disease-modifying anti-rheumatic drugs improve the cardiovascular profile in patients with rheumatoid arthritis

**DOI:** 10.3389/fcvm.2022.1012661

**Published:** 2022-10-24

**Authors:** Andrea Giachi, Massimo Cugno, Roberta Gualtierotti

**Affiliations:** ^1^UOC Medicina Generale Emostasi e Trombosi, Fondazione IRCCS Ca' Granda Ospedale Maggiore Policlinico, Milan, Italy; ^2^Department of Pathophysiology and Transplantation, Università Degli Studi Di Milano, Milan, Italy

**Keywords:** rheumatoid arthritis, cardiovascular disease, atherosclerosis, DMARDs, TNF inhibitors, JAK inhibitors, IL-6 inhibitors, methotrexate

## Abstract

Rheumatoid arthritis (RA) is a chronic inflammatory disease affecting about 0. 5–1% of the adult population and manifesting as persistent synovitis, systemic inflammation and production of autoantibodies. Patients affected by RA not only experience chronic disease progression, but are also burdened by a 1.5-fold increased cardiovascular (CV) risk, which is comparable to the risk experienced by patients with type 2 diabetes mellitus. RA patients also have a higher incidence and prevalence of coronary artery disease (CAD). Although RA patients frequently present traditional CV risk factors such as insulin resistance and active smoking, previous studies have clarified the pivotal role of chronic inflammation–driven by proinflammatory cytokines such as interleukin 6 (IL-6) and tumor necrosis factor alpha (TNF-alpha)–in accelerating the process of atherosclerosis and impairing the coagulation system. Over the last years, a number of studies have shown that disease-modifying anti-rheumatic drugs (DMARDs) reducing the inflammatory state in general improve the CV risk, however some drugs may carry some apparent negative effects. Thus, RA is a model of disease in which targeting inflammation may counteract the progression of atherosclerosis and reduce CV risk. Clinical and experimental evidence indicates that the management of RA patients should be tailored based on the positive and negative effects of DMARDs on CV risk together with the individual traditional CV risk profile. The identification of genetic, biochemical and clinical biomarkers, predictive of evolution and response to treatment, will be the next challenge for a precision approach to reduce the burden of the disease.

## Introduction

Rheumatoid arthritis (RA) is a chronic systemic inflammatory disease primarily affecting synovial joints, but it can also involve extra-articular organs such as skin, eye, lung, heart, kidney, digestive system, blood vessels, salivary glands, central and peripheral nervous systems, and bone marrow (1–3). The prevalence of the disease in western countries is estimated about 0.5–1% of the adult population, affecting about twice as many women as men, with a peak of incidence around the age of 50 years (3–6). Patients affected with RA not only experience chronic disease progression, but also are burdened by augmented morbidity and mortality due to CV disease (7–10); in particular, they have a higher incidence and prevalence of coronary artery disease (CAD) (11). Clinical and pre-clinical evidence support the pivotal role of chronic inflammation and endothelial dysfunction in accelerating the process of atherosclerosis, driven by proinflammatory cytokines such as interleukin 6 (IL-6) and tumor necrosis factor alpha (TNF-alpha) (12, 13). Over the last 10 years, substantial attention has been paid on the potential effects of different therapies in reducing CV risk, generating a plethora of academic work on this topic. Many studies have shown that treatment with disease-modifying anti-rheumatic drugs (DMARDs) improves not only clinical and laboratory measures of disease activity, but also CV outcomes, contributing to render the use of these therapies a cornerstone in RA treatment.

With this as background, we reviewed the evidence of the last 10 years on the interaction of inflammation with hemostatic and endothelial functions in RA, the underlying mechanisms, and the effects of different DMARDs in their reduction.

## Literature search

Here, we provide an overview on the effects of the most frequently prescribed DMARDs on cardiovascular risk in patients with RA in a narrative review. The following keywords were used in different combinations to identify relevant studies published in PubMed and Google Scholar before June 2022: “rheumatoid arthritis”, “biologics”, “biologic DMARDs”, “TNF-α antagonists”, “TNF inhibitors”, “anti-TNFs”, “anti-IL6”, “anti-IL1”, “JAK-inhibitors”, “canakinumab”, “tocilizumab”, “rituximab”, “abatacept”, “anakinra”, “etanercept”, “infliximab”, “adalimumab”, “atherosclerosis”, “cardiovascular disease”, “cardiovascular risk”, “myocardial infarction”, “heart failure”, “stroke”, “arterial stiffness”, “augmentation index”, “endothelial function”, “flow mediated dilatation”, “carotid”, “intima media thickness”. The search was limited to English-language publications only. We included articles regarding patients with rheumatoid arthritis. The selected articles were identified by specialists in rheumatology and internal medicine based on their expertise. Any articles for which no full text was available were excluded.

## Cardiovascular disease in patients with rheumatoid arthritis

CV disease in RA patients occurs in the form of premature development and accelerated progression of atherosclerotic lesions as well as hyperactivation of coagulation and endothelial dysfunction (14–17). All these mechanisms lead to a higher risk of CV complications, such as coronary artery disease, cerebrovascular disease and peripheral artery disease. In addition, they contribute to shortening the life expectancy in RA patients (18, 19). The prothrombotic state of RA patients may also involve the venous district, presenting as deep venous thrombosis and pulmonary embolism (20).

### Cardiovascular mortality

Patients with RA experience an increased mortality compared to the general population, however, over the last two decades, the long-term prognosis has improved significantly. Many studies demonstrated a drop in mortality for all causes since the early 2000s with a consistent trend in the following years. Up to a two-fold improvement in general standardized mortality rate, compared to the general population, has been observed from 2000 to 2017 (12, 21, 22).

Among different causes of mortality, nearly all studies agree on CV disease being the most common one, accounting for about 40 to 50% of deaths according to different reviews and meta-analyses based on studies from 1960 to 2008 (23–25). A recently published study, which analyzed 813 patients from Minnesota who were diagnosed with RA from 1980 to 2007, has found a 58% decline in CV mortality and 83% drop in coronary heart disease (CHD)-related mortality in patients receiving a diagnosis of RA from 2000 to 2007, compared to the 1990–1999 group (26). Consistently, an Australian study found an estimated standardized CV mortality rate of 1.2, substantially reduced compared to what was reported in earlier studies (27). In line with these findings, another study conducted on a low-disease activity RA cohort between 2009 and 2011 found a drop in CV case fatality compared to 1996 studies from 28.6 to 6.9% (28). This improvement in mortality is partially explained by the drop of mortality in the general population, but other factors such as early diagnosis, prompt treatment using novel molecules, an improved attention and intervention on other risk factors in RA patients have played a pivotal role in achieving a better prognosis.

### Cardiovascular morbidity

RA patients are burdened by many comorbidities such as infections, osteoporosis, gastrointestinal, pulmonary and hematologic diseases, however CV disease is the most frequent (4, 7). CV manifestations in RA are variable, affecting mainly the arterial system, which leads to myocardial infarction, stroke, peripheral artery disease and, to a lesser degree, angina (11). The risk of developing atherosclerotic CV disease in patients affected with RA is similar in magnitude to that of diabetic patients, being 1.5–1.6 fold higher than the general population (24).

A myocardial infarction risk of 68% more than the general population was described in a RA patients, according to a meta-analysis of observational studies, with no differences between men and women (25). A recently published population-based cohort study, which included patients diagnosed from 1980 to 2009, demonstrated a 56% decrease in acute myocardial infarction events between the group of patients who were diagnosed in the 2000s compared to the group of 1980s (hazard ratio [HR] 0.44) (29). Notably, the addition of the highest erythrocyte sedimentation rate (ESR) or time-dependent treatment with DMARDs into the predictive model showed a significant reduction of any CV event in the group of 2000s, supporting a correlation between inflammation and CV risk. More recently, in RA group compared to the general population, Yazdani et al. found an increased risk of acute myocardial infarction (RR 1.21), and Holmqvist et al. found an overall higher incident CV event rate (HR 1.41) (30, 31). These two studies, however, revealed similar rates of decline in myocardial infarction and CV events respectively for both RA and non-RA group over the course of the study period. To sum up, although it is unclear if the existing gap between RA patients and the general population in CV risk is really narrowing, available data consistently show a decrease in CV event rates in RA patients.

Concerning cerebrovascular accidents, a small number of studies is available, yet suggesting a burden similar to myocardial infarction, according to a meta-analysis that has demonstrated a 40% augmented incidence among RA patients (25). A recent German study confirmed this data, showing an association between RA and both stroke (HR 1.42, confidence interval [CI] 1.25–1.60) and transient ischemic attack (HR 1.69, CI 1.46–1.95) (32). Accordingly, a 2021 meta-analysis further supports a significantly increased risk of stroke in RA (HR 1.32 95%, CI 1.02–1.73), with no differences between male RA patients compared to controls, but with a higher incidence in the female RA subgroup (33).

Congestive heart failure may also occur almost twice as often in RA patients compared to the general population, as demonstrated by a recent meta-analysis (34). In the study by Khalid et al. it was also demonstrated that women affected by RA had a three-fold higher incidence of heart failure compared to controls (OR 3.38, 95%, CI 2.59–4.40), and the meta-regression showed an even greater incidence with older age (35).

Peripheral arterial occlusive disease resulted 1.73-fold higher (95% CI 1.57–1.91) in RA patients compared to a non-RA cohort, according to a study conducted on 30,812 patients with RA (36). Interestingly, the adjusted risk of peripheral arterial occlusive disease was more evident in patients with RA aged ≤ 49 years (HR 3.39, 95% CI 2.66–4.32). Another study assessing intima-media thickness in 80 RA patients without CV disease or diabetes demonstrated a higher prevalence of femoral intima-media thickness and plaques compared to controls matched for age, gender and CV risk factors (37).

The venous system is also involved in RA. According to a nationwide register-based cohort study, the relative risk for venous thromboembolism in RA is 1.88 (95% CI 1.65–2.15) and it increases with increasing RA disease activity, namely the 1-year incidence increases from 0.52% in patients in remission to 1.08% in subjects with high disease activity (38). These findings follow a 2014 meta-analysis that estimated a pooled risk of deep venous thrombosis, pulmonary embolism and overall VTE in patients with RA of 2.08 (95% CI 1.75–2.47), 2.17 (95% CI 2.05–2.31), and 1.96 (95% CI 1.81–2.11) respectively, compared to non-RA subjects (39).

## Cardiovascular risk factors in rheumatoid arthritis

### Traditional risk factors

Genetic factors play a role in determining CV disease in RA patients. Many single nucleotide polymorphisms are shared by both diseases and HLA DRB1^*^01 haplotype, which is linked to RA susceptibility, has been associated with C-reactive protein (CRP) levels and the risk of myocardial infarction in general population (40, 41).

In patients with RA, the study of incidence of traditional CV risk factors such as obesity, diabetes, hypertension and smoking gave contradictory results, with the only exception of smoking. Smoking has in fact been identified as a significant risk factor for both RA and CV risk (42), contributing to the appearance of anti-citrullinated c-peptide antibodies (CCP), increased expression of inflammatory genes promoting RA development, and methylation of genes involved in coronary artery disease (43–45). Among the aforementioned risk factors, it is also important to remember that in RA patients body mass index is inversely correlated with CV events (46); this is probably due to the chronic inflammatory state that causes a loss of lean muscle mass and accumulation of adipose tissue in a phenomenon known as rheumatoid cachexia (47). Moreover, lower mortality was observed in higher BMI patients with RA although the mechanism that protects these patients has not been explained (46).

Concerning lipid profile, in RA patients also exists a “lipid paradox”(48), meaning that there is no clear association between higher levels of low-density lipoprotein (LDL) and CV risk (48, 49), despite higher high-density lipoprotein (HDL) level being effectively associated with lower CV risk (49, 50). This is probably due to an impaired function of lipoprotein in the inflammatory state of RA patients, in the form of more oxidized LDL (51), which are more easily taken up by macrophages determining their transformation into foam cells, and pro-inflammatory HDL (52–54), which have a reduced capacity of reverse cholesterol transport (55).

Although often forgotten, RA patients are usually inactive (56). This is due to many factors, such as pain, lack of motivation and lack of knowledge about the impact of physical inactivity (57–59). In this sense, RA related disability is arguably an independent CV risk factor (60).

### Disease-specific risk factors

All the aforementioned risk factors, however, cannot fully explain the excess of CV disease mortality and morbidity in RA patients, thus suggesting RA itself being another independent risk factor (61). Mounting evidence, in fact, suggests the central role of disease activity and immune system in the pathogenesis of CV risk and it is nowadays known that proinflammatory cytokines such as IL-6 and TNF-alpha are implicated in both RA and atherogenesis (62). These molecules also have a role in contributing to insulin resistance (63) and have been independently associated with coronary artery calcifications (64), as well as CRP levels and swollen joint counts have been associated with carotid plaque progression (65).

A number of studies have demonstrated a correlation between higher disease activity and the risk of CV events (66, 67), evident since the early phases of the disease, with a 33% increase of CV disease risk every 1-unit increase of DAS28. Other studies have also successfully associated CRP and ESR levels with higher risk of CV disease (49, 68, 69). Various studies showed an association between disease activity and cardiac function, in particular demonstrating an increased left ventricular strain and left ventricular global longitudinal strain, diastolic dysfunction and left ventricular wall thickness (70–73), while another study demonstrated an improvement in cardiac function in RA patients in remission (74).

Another feature that is hypothesized to have a role in CV disease is the presence of specific autoantibodies. Anti-CCP and rheumatoid factor positivity have been associated with increased CV risk in both RA patients and non-RA patients, although not consistently (75–77). Two studies conducted by cardiac magnetic resonance in RA patients showed an association between anti-CCP antibodies and reduced stroke volume, end diastolic volume and left ventricular mass (78, 79).

Lastly, therapeutic intervention can also play a role in promoting CV disease risk. A meta-analysis demonstrated an 18% increased risk of CV events in RA patients treated with nonsteroidal anti-inflammatory drugs (NSAID) as well as a 47% increased risk in patients treated with glucocorticoids (80). With regard to glucocorticoids, there is a correlation between CV risk and both dose and duration of treatment (81–83). In the case of NSAIDs, this increased risk is probably due to a reduced production of prostacyclin that has a vasodilatory and antiplatelet effect, while glucocorticoids are well known to exacerbate other risk factors such as hypercholesterolemia, hypertension, hypertriglyceridemia and are also supposed to cause impaired reverse cholesterol transport of HDL (84, 85).

## Effects of different DMARDs on mechanisms of cardiovascular disease in rheumatoid arthritis

### Mechanisms of potential pharmacological target

**Figure 1:**
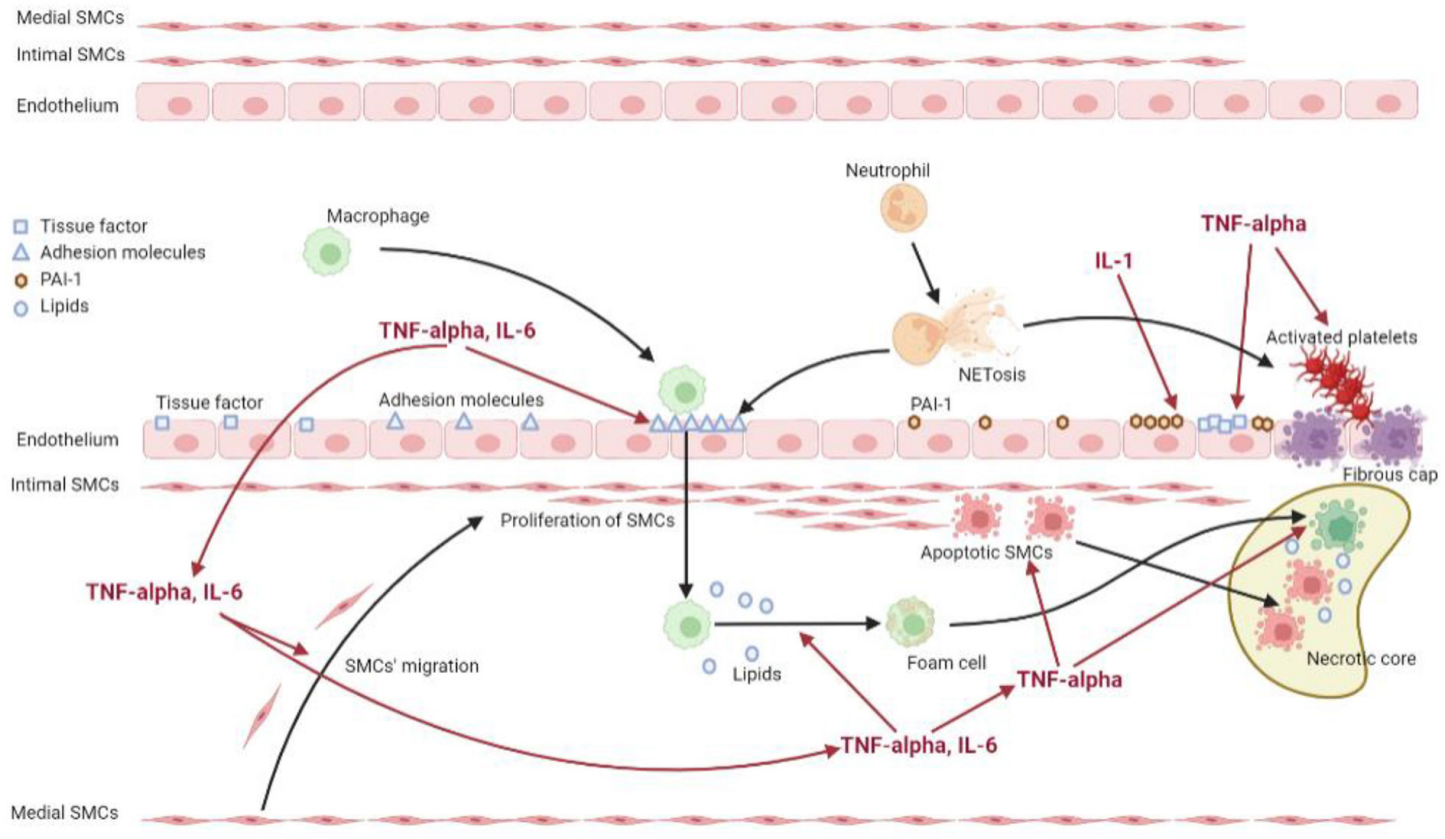
Mechanisms of inflammation promoting the atherosclerotic process in rheumatoid arthritis. The figure shows the interplay between inflammation and the different effectors of the atherosclerotic process. The systemic inflammation, supported by pro-inflammatory cytokines such as TNF-alpha, IL-6 and IL-1, acts on the endothelial cells inducing the expression of adhesion molecules, tissue factor and PAI-1. It also promotes the migration of smooth muscle cells from the media to the intima, as well as facilitates the uptake of lipids by the macrophages. Moreover, the inflammatory state acts on platelets and neutrophils, triggering the process of NETosis, which stimulates on one hand the expression of adhesion molecules by endothelial cells, on the other hand the activation of platelets contributing to a prothrombotic state.

The atherosclerotic process has been demonstrated to be particularly accelerated in RA patients (86). A relevant number of mediators and mechanisms are shared between the inflamed synovium and the atherosclerotic plaque, and in particular pro-inflammatory cytokines produced in the synovium contribute both directly and indirectly to plaque development (87, 88). In RA patients all the stages of atherosclerosis are amplified, including endothelial dysfunction, arterial wall inflammation, plaque formation, remodeling and CV events, which develop upon the rupture of the atherosclerotic plaque and the subsequent thrombosis (89). Figure 1 shows the mechanisms through which inflammation promotes the atherosclerotic process in RA.

Endothelial dysfunction is a condition characterized by the failure to perform its physiologic functions, such as regulation of vascular tone, cellular adhesion, vascular smooth muscle migration and resistance to thrombosis, often as a maladaptive response to a pathologic stimulus (90). It is characterized by upregulated expression of cellular adhesion molecules, reduction of the bioavailability of vasodilators, particularly nitric oxide (NO), and/or an increase in endothelium-derived contracting factors (91, 92). TNF-alpha expression has been demonstrated on the surface of microparticles promoting apoptosis and autophagy and contributing to endothelial dysfunction (93). Endothelial disfunction in RA patients, in fact, evaluated by brachial artery flow-mediated dilation, has been correlated to the level of inflammation by a meta-analysis of 20 studies (94). In addition, arterial stiffness, measured by pulse wave velocity or augmentation index, has been demonstrated to be higher in RA patients compared to controls (95). It is known that *in vitro* TNF-alpha addition stimulates superoxide release from endothelial cells and monocytes exacerbating the oxidation of LDL (96). These oxidized LDL are taken up by macrophages through scavenger receptors such as CD36, LOX-1 and SR-A, transforming macrophages into foam cells which lead to the formation of the fatty streak (97, 98). Expression of LOX-1 and SR-A results upregulated *in vitro* by TNF-alpha and IL-6 as well as CD36 expression is increased after exposure to the serum of a collagen induced arthritis animal model (99).

It has been demonstrated that in atherosclerosis of both RA and non-RA patients there is an increased expression of cell adhesion molecules such as ICAM-1 and P-selectin, although only P-selectin correlates with disease activity (90, 100–103). Moreover, in RA patients, and in particular in patients with extraarticular manifestation, several data have been reported showing an expansion of CD4+CD28- TH cells, a specific subset which could shift the immune response toward TH1 mediated activation of macrophages, leading to accelerated progression of atherosclerosis (104–106). Accordingly, a study conducted on aortic biopsies of patients undergoing coronary artery bypass graft surgery, showed a higher prevalence and density of medial and adventitial mononuclear cells infiltrates in patients with rheumatic disease such as RA compared to controls (107). TNF-alpha and IL-6 can also promote the proliferation and migration of vascular smooth muscle cells from the media to the intima, leading to intima-media thickening (108), and TNF-alpha is demonstrated also to activate platelets *in vitro* (109). This process culminates with the apoptosis of foam cells which generates a necrotic core rich in lipids, and the creation of a fibrous cap covering the plaque which can become unstable and rupture (110), leading to thrombosis which brings to CV events (111).

Regarding thrombosis, it is known that an extensive cross-talk between coagulation and inflammation pathways exists. Longitudinal studies have shown increased levels of markers for endothelial activation such as von Willebrand Factor, ICAM and VCAM in RA patients (112). TNF-alpha has been demonstrated to be able to upregulate *in vitro*, both in endothelial cells and in monocytes, the expression of tissue factor, which is the main initiator of blood coagulation through binding and activation of FVII (113, 114). Previous studies have also demonstrated that RA patients have higher levels of prothrombin fragment F1+2, a marker of thrombin generation, compared to healthy controls, and the infusion of TNF-alpha in healthy human volunteers can increase plasma concentration of this fragment (13, 115, 116). Fibrinolysis too is impaired in patients with RA. TNF-alpha and IL-1 simulate the production of plasminogen activator inhibitor-1 in endothelial cells, which counteracts the fibrinolytic system (117). Moreover, TNF-alpha and IL-1 are able to down-regulate thrombomodulin on the endothelial surface, disrupting also the protein C system (113). In addition, a relationship between platelet activation markers and RA has been demonstrated, and platelets have been suggested to amplify inflammation in RA through collagen-dependent microparticle production (118). Interestingly, these particles were increased also in synovial fluid of RA patients.

Another element that is supposed to have a role in atherosclerosis of RA patients is represented by the neutrophils, and in particular by neutrophil extracellular traps (NETs), structures consisting of DNA, histones and neutrophil-derived granule proteins, which are expelled from neutrophils and boost the adaptive response of dysfunctional T- and B-cells (119, 120). In RA patients, neutrophils are more prone to undergo NETosis, and the citrullinated histones within the NET are a leading substrate for the generation of anti-citrullinated protein autoantibodies (121–124). NET components are also supposed to act as damage-associated molecular patterns and sustain a vicious circle leading to endothelial expression of adhesion molecules and pro-thrombotic factors such as von Willebrand factor and P-selectin, as well as digest tissue factor pathway inhibitor and increase platelets responsiveness (125–128). Accordingly, the content of NETs in coronary thrombectomy specimens positively correlates with infarct size in the general population (129).

To sum up, the systemic inflammation of RA patients on one hand promotes and accelerates the different steps of atherosclerosis, on the other hand generates a prothrombotic state that increases the risk of CV events. For these reasons, DMARDs, reducing inflammation, can counteract at various steps the determination of CV events and therefore could have a role in reducing the CV risk of RA patients.

### Available anti-rheumatic drugs

The different types of DMARDs commonly used in RA patients are classified based on their process of discovery and mechanistic distinctions in conventional synthetic DMARDs (csDMARDs), biologic DMARDs (bDMARDs) and targeted synthetic DMARDs (tsDMARDs) (130). Table 1 resumes the main effects of the most commonly used DMARDs on CV risk.

**Table 1:** Disease-modifying anti-rheumatic drugs (DMARDs) commonly used in patients with rheumatoid arthritis and their potential effects on cardiovascular risk.

**Drug**	**Type**	**Mechanism of action**	**Mode of administration**	**Positive effects on CV risk**	**Negative effects on CV risk**	**References**
Methotrexate	csDMARD (small molecule)	Dihydrofolate reductase inhibitor	Oral, subcutaneous, intramuscular	Increased adenosine production, increased reverse cholesterol efflux capacity, effect on endothelium by induction of the mitochondrial anti-oxidant manganese superoxide dismutase.	Induction of hyperomocysteinemia (concomitant folate therapy is recommended)	(131–135)
Adalimumab	bDMARD (mAb)	TNF-inhibitor	Subcutaneous	Inhibitory effect on cholesterol uptake in macrophages, reduced expression of vascular adhesion molecules on endothelial cells, increased total HDL, reduction of apolipoprotein B/A, reduction of aortic stiffness, reduction of the progression of subclinical carotid atherosclerosis	Increased total triglycerides	(52, 136–139)
Infliximab	bDMARD (mAb)	TNF-inhibitor	Intravenous	Reduction of plasminogen activator inhibitor-1, increased total HDL, reduction of apolipoprotein B/A, reduction of aortic stiffness, reduction of the progression of subclinical carotid atherosclerosis	Increased total triglycerides	(117, 136, 138, 139)
Etanercept	bDMARD (fusion protein)	TNF-inhibitor	Subcutaneous	Improvement in left ventricular mass index, increased total HDL, reduction of apolipoprotein B/A, reduction of aortic stiffness, reduction of the progression of subclinical carotid atherosclerosis	Increased total triglycerides	(136, 138–140)
Tocilizumab	bDMARD (mAb)	IL-6 receptor inhibitor	Intravenous	Reduction of lipoprotein(a) levels, reduction of apolipoprotein-A expression, increased cholesterol efflux capacity of HDL.	Increased levels of LDL	(141–143)
Anakinra	bDMARD (mAb)	IL-1 receptor antagonist	Intravenous	Improvement of left ventricular longitudinal strain, improvement of coronary flow reserve, reduction of glycated hemoglobin levels.	-	(144, 145)
Rituximab	bDMARD (mAb)	Anti CD20	Intravenous, subcutaneous	Increase levels of HDL and apolipoprotein B/A1 ratios.	-	(146–148)
Abatacept	bDMARD (fusion protein)	Block of CD80 and CD86	Intravenous	Improvement of insulin resistance (more evident in diabetic patients)	-	(149–152)
Tofacitinib	tsDMARD (small molecule)	JAK inhibitor	Oral	Immunomodulatory effect on proinflammatory cytokines, increased HDL levels, reduction of platelet activation	Increased serum LDL and triglycerides	(153–156)

CV, cardiovascular; DMARD, disease-modifying anti-rheumatic drug; csDMARD, conventional synthetic DMARD; bDMARD, biologic DMARD; tsDMARD, targeted synthetic DMARD; mAb, monoclonal antibody; HDL, high-density lipoprotein; LDL, low-density lipoprotein; TNF, tumor necrosis factor; IL, interleukin; JAK, Janus kinase.

#### Methotrexate

Methotrexate is an anti-metabolite inhibitor of the enzyme dihydrofolate reductase, considered for long time a cornerstone of RA treatment for its immunomodulating properties when administered in a low-dose regimen. In a systematic review and meta-analysis of 28 cohort studies (80), methotrexate was associated with a pooled 28% reduction in CV events, as well as other studies have even increased this correlation up to 34–66% (157, 158). More recently, an updated meta-analysis confirmed these findings (159). The effect on atherosclerotic lesion progression is not clear, as a number of studies has demonstrated an improvement of atherosclerosis after methotrexate therapy, although other authors didn't confirm these findings; due to the limited number of studies and the small sample sizes, it is still not fully disclosed (65, 160–165). In 2019, the CIRT study investigated the potential protective effect of low-dose methotrexate in CHD patients without RA, but it failed to prove it; between possible explanations, it is notable that the mean CRP levels of this study population (1.6 mg/L) was lower than both the median CRP levels of RA patients, as reported by Ajeganova et al. (131), and the residual inflammatory risk in CHD as currently defined (2 mg/L). The effects through which methotrexate reduces CV event rates in RA patients are several and still not fully understood; it is however known that the clinical response to treatment is associated with reduction of inflammation and reduction in the expression of inflammatory cytokines such as TNF-alpha and IL-6, which, as aforementioned, have proatherogenic effects. Emerging evidence suggests that methotrexate can also induce adenosine production, which decreases the activation of lymphocytes by binding the adenosine A2 receptor (132), and increases the reverse cholesterol efflux capacity through both adenosine production and ATP-binding cassette transporter A1 expression. Moreover, it has been demonstrated *in vitro* that methotrexate exerts a direct effect on endothelium by inducing the mitochondrial anti-oxidant manganese superoxide dismutase (MnSOD) and scavenging superoxide radicals actively (133, 134). Finally, it is interesting to notice that methotrexate has an overall cardioprotective effect in RA patients even though it is known to induce hyperomocysteinemia, which is arguably atherogenic (135). Concomitant folate supplementation has been shown to decrease the plasma homocysteine level and consequently may protect against CV risk (166).

#### TNF inhibitors

According to the meta-analysis reported above (80), a 30% reduction of risk of CV events exists in RA patients treated with TNF inhibitors. In particular, among the studies with MI or stroke as outcome, the use of TNF inhibitors was associated with a 41% relative risk reduction of MI and a 43% reduction of stroke. However, these effects may not be found in all patients receiving TNF inhibitors, rather seem to be related to clinical response, as some studies have observed a reduction of CV events only in good responders to anti-TNF treatment (136, 167). Among the mechanisms of action, many of which have been explained in previous sections, TNF inhibitors improve endothelial dysfunction, oxidative stress and modify the lipid profile. A meta-analysis of 13 studies has demonstrated in long term anti-TNF users an increase of total HDL and triglycerides, as well as stable levels of LDL and reduction of apolipoprotein B/A (137). An *in vitro* study with TNF inhibitor adalimumab demonstrated an inhibitory effect on cholesterol uptake in macrophages, without affecting reverse cholesterol transport (53), while another *in vitro* study, always with adalimumab, showed a reduced expression of vascular adhesion molecules on endothelial cells (140). Finally, treatment with infliximab has been associated with a significant reduction in quantity and activity of tissue-type plasminogen activator (tPA) and plasminogen activator inhibitor-1 (PAI-1), conditions showing a prothrombotic effect (117). Treatment with TNF inhibitors has been associated with improvement in left ventricular mass index after 3–6 months of treatment alongside clinical improvement (138), reduction of aortic stiffness after 3 months (139), and reduction of the progression of subclinical carotid atherosclerosis after 14 weeks (168). The TARGET trial, a current phase 4 study which aims to compare post-RA treatment changes in aortic and carotid inflammation, quantified through PET-CT analysis, will be the first interventional study in RA trying to provide insight on the effects of DMARDs on a direct measure of vascular inflammation (169).

#### IL-6 inhibitors

Tocilizumab is a humanized monoclonal antibody antagonist of the IL-6 receptor. Therapy with tocilizumab is approved for the treatment of patients with moderate or severe RA, both in combination with conventional DMARDs and in monotherapy. In RA patients, tocilizumab showed to be effective in improving clinical symptoms (170) and reducing both inflammatory and prothrombotic biomarkers (171, 172). Moreover, tocilizumab may improve the pro-atherothrombotic status of RA patients by regulating the inflammatory activity of monocytes and neutrophils through mechanisms involving modulation of oxidative stress, NETosis, and intracellular signaling (141). Despite the increase in LDL levels observed during treatment with tocilizumab, the incidence of CV events seems reduced as compared to RA-population in an observational study (173). Given these effects, the ENTRACTE study, a phase IV study (142) with an over 3 years follow-up comparing tocilizumab and etanercept, a TNF inhibitor, showed that the reduction of CV risk of tocilizumab is comparable to that of etanercept. This may be in agreement with the observation that IL-6 blockade is able to induce a reduction in lipoprotein (a) [Lp(a)] levels (174), a reduction of apolipoprotein-A expression and the inhibition of IL-6-induced Lp(a) mRNA expression (143). Consistently, a recent study demonstrated that treatment with tocilizumab in RA patients reduces pro-atherogenic effects of LDL and increases the cholesterol efflux capacity of HDL (175). Sarilumab is another antagonist of IL-6R. It is a fully human monoclonal antibody against IL-6R successfully used to control symptoms in RA patients (176). To the best of our knowledge, the only study on CV effects of sarilumab is that of *Fleischmann et al.*, which showed a small reduction of incidence of major CV events (from 1.4 to 0.5 per 100 patient-years) compared to general RA population (144).

#### IL-1 inhibitors

Among the available IL-1 inhibitor drugs, only anakinra, a human recombinant IL-1 receptor antagonist, is currently approved for RA treatment. Thus, only a few studies exist and to our knowledge no one has ever estimated the effect of anakinra or other IL-1 inhibitors on CV risk in RA patients. One study has demonstrated an improvement of cardiac function and a reduction of endothelial dysfunction, measured by left ventricular longitudinal strain and coronary flow reserve respectively, and another has reported an improvement of glycemic control, assessed by glycated hemoglobin levels (145, 177).

Interestingly, canakinumab, another IL-1 inhibitor, has demonstrated a reduction of the rate of CV events compared to placebo in a population of patients not affected by RA but by recurrent CV events and high levels of inflammatory markers (146).

#### Rituximab

There are few data regarding the effects of rituximab therapy on atherosclerosis in RA patients. Small short-term studies demonstrated an improvement in atherogenic index, along with increased levels of HDL and apolipoprotein B/A1 ratios, although these effects were restricted to clinical responders, thus suggesting that the main promoter of these modifications is the reduction of inflammation more than the direct effect of the drug (147, 148, 178). The CORRONA registry, which investigated CV events rates in RA patients matched by participant characteristics, showed comparable efficacy between the group treated with rituximab and the one treated with TNF inhibitors (179).

#### Abatacept

Abatacept is a fusion protein consisting of cytotoxic T-lymphocyte antigen-4, an anti-inflammatory factor expressed by T cells, conjugated to the Fc portion of IgG, which has demonstrated clinical efficacy in RA (149). Only a small handful of studies exists: two small studies on aortic stiffness in RA patients which initiated abatacept treatment did not demonstrate an improvement, while a cohort study comparing patients that initiated therapy with abatacept or TNF inhibitors showed a modest reduction of CV risk in the group treated with abatacept, particularly in patients affected by diabetes mellitus (150–152, 180). Studies conducted on murine models, however, demonstrated a reduction in atherogenesis in mice treated with abatacept (181, 182).

#### JAK inhibitors

Janus Kinase (JAK) inhibitors are the most recent class of drugs approved for RA treatment. The JAK family is a family of receptor tyrosine kinases that, when attached to a specific cytokine, act as dimers and phosphorylate, binding signaling peptides of the STAT family, which eventually translocate in the nucleus in order to regulate the transcription of target genes. This pathway ends up regulating the expression of numerous cytokines, such as IFN, IL-4, IL-6 and IL-10, which are included in different immunological pathways and in pathogenesis of RA. Currently, four different JAK inhibitors are licensed for RA treatment in western countries: tofacitinib, baricitinib, upadacitinib and filgotinib, all demonstrating their efficacy in treatment of RA (183, 184).

During the development phase of tofacitinib, however, a slight increase in the incidence of cancers, including lymphoma, was observed, as well as an increase in serum lipid levels (153, 154). This led to a prospective safety trial comparing tofacitinib with TNF inhibitors, the Oral Rheumatoid Arthritis Trial (ORAL) Surveillance. According to this study, the incidence of major CV events was higher in the tofacitinib group (3.4%) compared to TNF inhibitors group (2.5%), the most common of which being nonfatal myocardial infarction for tofacitinib and nonfatal stroke with TNF inhibitors, in particular for patients 65 years of age or older (155). This study showed also an increased risk of venous thromboembolism with the tofacitinib 10-mg dose, further enhancing the doubts about safety raised by a previous trial with 4-mg dose baricitinib, even though it was not powered to this specific evaluation. The same study showed a similar risk of venous thromboembolism between 5-mg dose tofacitinib and TNF inhibitors, a finding in line with real-world data from the CORRONA registry (185). The 2022 STAR-RA population-based study, which evaluated Medicare data on patients which initiated treatment with tofacitinib or TNF inhibitors, showed no difference in the incidence rates of myocardial infarction and stroke between the two groups, even when the analysis was restricted to patients with similar CV risk factors to those of the patients enrolled in the ORAL Surveillance study (156). A biological explanation for the better effect on arterial thrombosis may be provided by the study of Parra-Izquierdo et al. that showed an inhibitory effects of JAK inhibitors on platelet function (186).

## Cardiovascular risk management

As reported above, the use of conventional synthetic and biological disease-modifying agents is associated with reduced CV risk in individuals with RA. These aspects on influence of anti-rheumatic drugs on CV involvement in RA, have been recently reviewed by Atzeni et al. (187) and subsequently by the recently published ORAL Surveillance and STAR-RA studies (155, 156).

From a practical point of view, the best possible approach should consider the assessment of the CV risk according to the current predictive scores such as Systematic Coronary Risk Evaluation (SCORE), Framingham score and QRISK (188, 189). The European League Against Rheumatism (EULAR), in their most recent guidelines on CV management in RA patients that date back to 2015, recommends to apply a 1.5 multiplication factor in order to adjust for the augmented risk of RA patients if the algorithm does not take it into account (190). The European Society of Cardiology (ESC) suggests to use the same adjustement for CV assessment (191). Indeed, available calculators have in some cases already included this adjustement: the SCORE calculator considers a 1.5 multiplication factor for RA patients, while QRISK calculator uses a 1.2 factor. According to the same EULAR guidelines, the assessment of CV risk in RA patients should be performed at least every 5 years if the risk is low to moderate, but we suggest to do it at least once per year in patients with moderate to high CV risk (SCORE >5%). Notably, both SCORE and Framingham score have been demonstrated to be unreliable in identifying patients at a high risk of atherosclerosis in two black African cohorts, thus demonstrating that the path leading to an effective score is still long (192, 193).

Other elements that must be taken into account are the family history of the patient, sex, personal history of CV disease, psychosocial factors, drug history, alongside assessment of lipid levels and biomarkers of inflammation and thrombosis. As aforementioned, it is important to remember that many commonly used DMARDs have an effect on lipid levels that should not be overlooked, as well as the achievement of a stable control of disease activity should be obtained in order to limit the confounding effects of systemic inflammation.

Imaging evaluation using echocardiography, arterial augmentation index or aortic pulse wave velocity have been proposed to further evaluate the patients, especially those with high CV risk, besides the current EULAR recommendations, which only include carotid ultrasound.

Regardless of risk level, however, all patients affected by RA should receive extensive lifestyle recommendations which must include, but not be limited to, regular exercise, healthy diet and smoking cessation. In addition, management of conditions such as dyslipidemia or hypertension should not differ from general population, although further studies are needed in order to assess the appropriateness of current treatment targets in RA patients.

To date, the approach based on the aforementioned data provides tools that can be used in a multidisciplinary discussion with other specialists involved in the evaluation of CV risk (i.e., cardiologists, internists, diabetologists and other specialists).

In the future, researchers will need to collect a huge series of genetic, serological, biochemical and diagnostic imaging data, to set up new tools for health stakeholders in order to put in practice a true personalized medicine in RA, as previously done in other fields such as oncology (194).

## Conclusions

Evidence has been provided supporting the view that the inflammatory state of RA patients contributes to accelerated atherosclerosis and to thrombosis, inducing several predisposing factors such as insulin resistance, dyslipidemia, endothelial dysfunction, as well as activation of coagulation and inhibition of fibrinolysis.

Over the last decades, a growing understanding of the complex pathogenesis of RA has led to a significant improvement in the management of the disease. DMARDs have been demonstrated to act on the inflammatory state, on one hand reducing the tissue damage and endothelial dysfunction locally, on the other hand lowering the systemic CV and atherothrombotic risk. Although some DMARDs may negatively affect specific CV risk factors, they show a net CV protective effect, mainly due to their overall anti-inflammatory activity. Therefore, RA can be considered a prototype example showing the importance of targeting inflammation in order to interfere with the atherosclerotic process and reduce CV risk.

Biologic DMARDs reduce the inflammatory process through different mechanisms and interfere with other systems, including lipid metabolism. In particular for tocilizumab, its detrimental effect of increasing LDL is counterbalanced by the improvement of HDL cholesterol efflux capacity. Concerning conventional synthetic DMARDs, almost all authors agree that responders to methotrexate have an improvement of CV profile, consistent with the reduction of inflammation and a direct effect on endothelial dysfunction. Targeted synthetic DMARDs, like JAK inhibitors, have shown efficacy in controlling RA activity although more recent data seem to suggest a higher risk of cancer and CV events compared to TNF inhibitors.

In this scenario, further research is needed to fully clarify the exact mechanisms through which RA promotes atherosclerosis and how DMARDs may counteract it. Furthermore, the recent findings on the adverse effects of JAK inhibitors raise the urge for long follow-up studies, involving large numbers of patients, in order to resolve the controversies on their safety and efficacy.

The main challenge for the future will be to develop a personalized approach for RA patients based on genetic, biochemical, clinical and radiological parameters, in order to identify the most appropriate therapeutic option for each patient in terms of clinical response, improvement of CV profile and reduction of adverse events. Personalized medicine in RA will overcome the actual “trial and error” approach that leads to a higher burden for patients and healthcare.

Considering the CV burden of RA patients, which is still high, it is crucial for the clinician to tackle the traditional CV risk factors following guidelines for the general population, and to tailor a patient-based therapy that considers the positive and negative effects of different drugs also on CV risk.

## Author contributions

All authors listed have made a substantial, direct, and intellectual contribution to the work and approved it for publication.

## Funding

This research was partially supported by Ricerca Corrente 2022 - Italian Ministry of Health, Cariplo and Fondazione Regionale per la Ricerca Biomedica (FRRB) n. 2017–1938.

## Conflict of interest

The authors declare that the research was conducted in the absence of any commercial or financial relationships that could be construed as a potential conflict of interest.

## Publisher's note

All claims expressed in this article are solely those of the authors and do not necessarily represent those of their affiliated organizations, or those of the publisher, the editors and the reviewers. Any product that may be evaluated in this article, or claim that may be made by its manufacturer, is not guaranteed or endorsed by the publisher.
